# Effects of Ascorbic Acid and β-1,3-Glucan on Survival, Physiological Response and Flesh Quality of Cultured Tiger Grouper (*Epinephelus fuscoguttatus*) during Simulated Transport in Water

**DOI:** 10.3390/biology9020037

**Published:** 2020-02-21

**Authors:** Bo Wu, Qi Wang, Jie Cao, Jun Mei, Jing Xie

**Affiliations:** 1 College of Food Science and Technology, Shanghai Ocean University, Shanghai 201306, China; m170200511@st.shou.edu.cn (B.W.); m190310920@st.shou.edu.cn (Q.W.); m190300743@st.shou.edu.cn (J.C.); 2 National Experimental Teaching Demonstration Center for Food Science and Engineering, Shanghai Ocean University, Shanghai 201306, China; 3 Shanghai Engineering Research Center of Aquatic Product Processing and Preservation, Shanghai 201306, China; 4 Shanghai Professional Technology Service Platform on Cold Chain Equipment Performance and Energy Saving Evaluation, Shanghai 201306, China

**Keywords:** *Epinephelus fuscoguttatus*, simulated transport in water, environment stress, β-1,3-glucan, physiological responses

## Abstract

Transport in water is the most common method for achieving high survival rates when transporting cultured fish in China; yet, transport success relies on proper water quality and conditions. This research was designed to explore the effects of ascorbic acid and β-1,3-glucan on survival, physiological responses, and flesh quality of farmed tiger grouper (*Epinephelus fuscoguttatus*) during simulated transport. The transport water temperature for live tiger grouper was 15 °C, which had the highest survival rate, the lowest stress response, and metabolic rate, and this will reduce the susceptibility to diseases. It is stated that β-1,3-glucan influences the changes of cortisol content, heat shock protein 70, IL-1β, and IgM transcription levels during simulated transport. Rather than using ascorbic acid alone (the A-group), β-1,3-glucan (3.2 mg/L) in the presence of ascorbic acid (25 mg/L) can effectively reduce the increase of transport-induced serum cortisol content, heat shock protein 70, and IL-1β, but stimulated IgM. 25 mg/L ascorbic acid and 3.2 mg/L β-1,3-glucan had no obvious effect on the nutritional indexes and flavor of live tiger grouper; however, these can effectively reduce the stress response, improve the innate immune activity, and ensure a higher survival rate.

## 1. Introduction

Aquaculture is a rapidly growing industry in the world, providing one of the most sustainable forms of edible protein and nutrient production [1]. According to a report by FAO in 2014, the global aquaculture production has doubled in the past decade and it now accounts for about 50% of fishery products. China produced 41.1 million tons of farmed food fish in 2012, contributing to approximately 61.7% of total world production [2]. High demand for fish is due to an increased consumer awareness of healthy food [3]. These increases have occurred in some commercially important species, such as grouper, seabass, rainbow trout, tilapia, large yellow croaker, and catfish. Live fish species are sold at a higher price than frozen ones. Therefore, research regarding the handling and transport of live fish to improve the viability of commercial fish is essential.

Currently, groupers are cultured on an industrial scale in Asian countries, because of their fast growth, efficient feed conversion, and good flesh quality [4,5]. Tiger grouper (*Epinephelus fuscoguttatus*) is known for its high protein, low fat, tender meat quality, and good taste, making it one of the most cultured grouper species in China, Japan, and Singapore [6,7].

Fish transport in China is not efficient in terms of technology and cost. It is required to develop the transport technology to produce a higher viability of commercial fish at a rational price. Transport is a strong stressor [8]. The stress intensity depends on transport conditions, such as package density and transport-water quality [9,10]. Environmental stressors could lead to a reduced immune system function, resulting in sickness and death [11,12,13,14]. Besides, fish were continuously stressed during fish transport, which resulted in quality deterioration [15,16]. Conversely, anesthesia could reduce the metabolic rate, oxygen demand, and response to stress [17,18], which enables fish to be more efficiently transferred in higher densities. Anesthesia has been used to handle and transport live fish for years to reduce stress on fish [19,20,21]. However, it is forbidden to use in the fish transport in China because of safety issues. 

Low temperature dormancy, while using anti-stress and immunopotentiator agents, are common practices to relieve stress on fish transport [22,23]. Low temperature dormancy can reduce mechanical damage, energy consumption, and stress response during fish transport [24,25]. However, an unsuitable low temperature could lead to a low survival rate [26,27]. Anti-stress agents include bioactive polysaccharides, vitamins, amino acids, mineral elements, and electrolytes [28,29]. Ascorbic acid is not only a good anti-stress agent, but it is also an effective immunostimulant [30]. Cheng et al. [31] reported that ascorbic acid could protect against DNA damage, apoptosis, and proteolysis of pufferfish under low temperature stress. The use of immunostimulants from various sources, such as fungi, algae, and bacterial products, is a common practice in aquaculture to strengthen the immune system of the cultured fish [32]. β-1,3-glucan is a polysaccharide that is commonly used as feed additive in aquaculture. Feeding with β-1,3-glucan might enhance innate immune responses, which can reduce the inhibition of the immune system that is caused by glucocorticoids and steroidal corticosteroids that are secreted by fish under stress [33,34]. Lin et al. [35] showed that β-1,3-glucan, chitosan, and raffinose could enhance the immune responses of koi. Anti-stress agents and immunostimulants can improve the antioxidant capacity and immunity of fish during transport, thereby improving the survival rate [36,37]. Therefore, the objective of this study was to investigate the effects of ascorbic acid and β-1,3-glucan on survival, physiological responses, and flesh quality of cultured tiger grouper during fish transport.

## 2. Materials and Methods

### 2.1. Preparation of Tiger Grouper

The live cultured tiger grouper (500 ± 50 g) were purchased from a local market in Luchao Port town (Shanghai, China), and were then transported to the laboratory while using a truck that was equipped with an insulated tank. The fish were kept in a prepared polyethylene tank (2.4 × 1.7 × 0.6 m) for two days before the experiment, to allow them to adapt to the experimental environment, where the average water temperature was 27 °C, water tank salinity was 26‰, the mean pH was 7.0, and the average dissolved oxygen was 6.0 mg∙L^−1^.

### 2.2. Experimental Design

#### 2.2.1. Experiment 1: Transport Temperature Determination

After the tiger grouper had acclimated for two days, the water temperature was adjusted at a rate of 2 °C/h from room temperature to 10, 13, 15, 18, 21, 24, 27, and 30 °C, respectively [38]. Subsequently, each fish was packed in a plastic bag with an equal weight of water, and filled with oxygen. Transport of fish was simulated in a vibration conveyor under 70 rpm (LX-100VTR, Shanghai Luxuan Instrument Equipment Factory, Shanghai, China). The survival rate was recorded, and fish were sampled at 0, 3, 10, 17, 24, 48, and 72 h after transport. Three fish were sampled in each group at each time. Survival time of each fish was also recorded, and mean values of these parameters were calculated to determine the transport temperature for the subsequent experiments. Each transport group had 20 fish and the total number of fish was 150. 

#### 2.2.2. Experiment 2: Anti-Stress Agent Exposure

β-1,3-glucan (2.4, 3.2, and 4.0 mg/L, respectively, Aladdin Biochemical Technology Co., Ltd., Shanghai, China), and 25 mg/L ascorbic acid (Aladdin Biochemical Technology Co., Ltd., Shanghai, China) were prepared. Afterwards, each fish was packed in a plastic bag with an equal weight of prepared water (fish-to-water ratio was 1:1). Moreover, oxygen was added to transport bags, and the content of oxygen reaches more than 80%. Table 1 shows the experimental treated samples. The transport of fish was simulated in a vibration conveyor under 70 rpm at 15 °C for 24 h. Three fish samples were randomly selected in each group at each time for analysis on 0 and 24 h during simulated transport and 48 h after recovery, respectively. The blood and liver of tiger grouper samples were used to determine the physiological and biochemical indicators, such as stress response, and muscle tissue was used to measure nutritional characteristics, taste, and flavor.

### 2.3. Serum Cortisol Assessment

Serum cortisol was measured by cortisol ELISA kits (Jiancheng Biological Engineering Institute, Nanjing, China), while following the manufacturer’s instructions.

### 2.4. Analysis of Enzymatic Activity

#### 2.4.1. Metabolic and Antioxidant Enzyme Activities

Acid phosphatase (ACP-A060-2-2), alkaline phosphatase (AKP-A059-2-2), and glutathione reductase (GR-A062-1-1) of serum were analyzed by corresponding kits (Jiancheng Biological Engineering Institute, Nanjing, China), following the manufacturer’s instructions.

#### 2.4.2. Immunological Enzyme Activity

Lysozyme (LZM-A050-1-1) was analyzed by using commercial analysis kits (Jiancheng Biological Engineering Institute, Nanjing, China), following the manufacturer’s instructions.

### 2.5. Real-Time PCR

Relative expression levels of HSP70, IgM, and IL-1β were determined by RT-PCR, as described by Lee et al. [39]. The total RNA from liver tissues was extracted with RNA rapid extraction kit (TaKaRa Biological Engineering Co., LTD., Dalian, China), quantified, and spectrophotometrically assessed for purity. RNA was then treated with DNase I (TaKaRa Biological Engineering Co., LTD., Dalian, China) to remove gDNA contamination, and complementary DNA (cDNA) was synthesized with M-MuLV reverse transcriptase. RT-PCR analyzed the expression levels of the selected immune-related genes. This was done in a 10 μm total volume while using SYBR Green I chimeric fluorescence, and with 500 nmol primers. PCR cycling conditions for all genes were, as follows: 94 °C for 10 min., 45 cycles at 95 °C for 30 s, 60 °C for 30 s and 72 °C for 30 s, followed by 10 min. at 72 °C. Relative expression levels of the target genes transcript (HSP70, IgM, and IL-1β), with GAPDH as an internal control, were calculated using a CFX manager software version 2.0 (Bio-Rad). Table 2 shows the primers used. The threshold cycle (Ct) values were obtained from each sample after finishing the program.

### 2.6. Biochemical Analysis

#### 2.6.1. Chemical Composition of Muscle

Ash, fat, moisture content, and total protein were measured according to Ayanda et al. [40].

#### 2.6.2. Serum Biochemical Testing

Creatine kinase (CK), uric acid (UA), total protein (TP), albumin (ALB), urea, and creatinine were assessed according to Jia et al. [41].

### 2.7. Nucleotides

The nucleotide extracts were prepared based on the method of Fang et al. [42]. ATP-related compounds, including inosine monophosphate (IMP), inosine (HxR), and hypoxanthine (Hx), were analyzed while using HPLC (Waters 2695, Milford, MA, USA), equipped with a VP-CDS C18 column (150 × 46 mm). 0.05 M phosphate buffer solution (pH 6.7) was used as the mobile phase. The flow rate was 1 mL/min., and the injection volume was 10 μL. The peak was detected at 254 nm. 

The taste activity value (TAV) was calculated as the following equation:

$$TVA=\frac{C}{T}$$

in which C corresponds to the absolute concentration of taste substances, mg/100 g, and T reflects the taste threshold, mg/100 g, (IMP: 25 mg/100 g, AMP: 50 mg/100 g).

### 2.8. Free Amino Acids (FAAs) Assessment

5 g mashed tiger grouper muscle tissue sample and 15 mL of 15% cold trichloroacetic acid were mixed and homogenized at 10,000 rpm, for 5 min. After standing at 4 °C for 2 h, the homogenate was centrifuged at 5980× *g* for 15 min, at 4 °C. Next, 5 mL supernatant was immediately neutralized to pH 2.00 and then diluted to 10 mL with ultrapure water [43]. The mixture was then filtered through a 0.22 μm, and an amino acid analyzer determined the contents of FAAs (Hitachi L-8800, Tokyo, Japan). 

### 2.9. Statistical Analysis

All of the assumptions were met prior to data analysis. Data were expressed as the mean ± SD and the one-way analysis of variance (ANOVA) procedure followed by Duncan’s multiple range tests was adopted to determine the significant difference (*p* < 0.05) between treatments.

## 3. Results and Discussion

### 3.1. Pre-Experiment: Selection of Tiger Grouper Transport Temperature and Ascorbic Acid Addition

Tiger groupers were transported at 10, 13, 15, 18, 21, 24, 27, and 30 °C. Survival rates that were recorded during simulated transport are presented in Table 3. Survival time of tiger grouper at 10 °C was less than 3 h. However, temperatures at 15, 18, 21, 24, and 27 °C could extend the survival time, and the survival rates were higher than 75%. Therefore, those transport temperatures were recommended for further experiments.

### 3.2. Effect of Temperature on Stress Responses of Tiger Grouper during Simulated Transport

**Figure 1:**
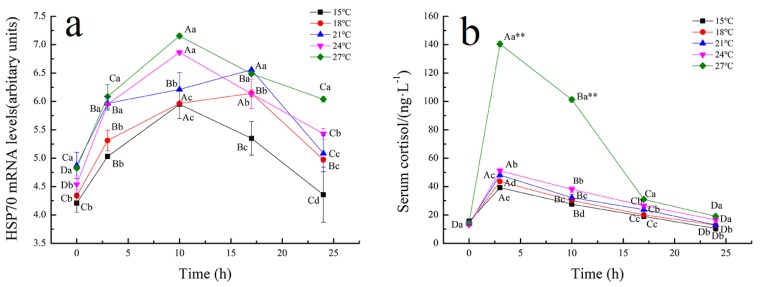
Effects of temperature on HSP70 (**a**) and serum cortisol (**b**) of tiger grouper during simulated transport. Among different temperature transport groups, different small and capital letters indicate the results of Duncan’s test at different transport time. The same letters mean no significant difference (*p* > 0.05), while different letters mean significant difference (*p* < 0.05). Different letters without * = *p*-value < 0.05, different letters with ** = *p*-value < 0.01.

HSP is a protein that will respond to external stressful conditions [44]. It protects cells from extreme physiological, pathological, and environmental conditions, and plays a role in protein misfolding correction, preserving immature polypeptides from aggregation under stress [45,46]. Cortisol is the main glucocorticoid hormone in teleosts that are involved in the regulation of metabolic adjustments. Under stress conditions, the increase of plasma cortisol promotes protein, glucose, and lipids mobilization in the skeletal muscle, which provide energy to overcome the stress [47]. The transcriptional level of HSP70 in the liver of all tiger grouper samples, at different transport temperatures, increased to the maximum value at 10 h, and then decreased, as shown in Figure 1a. Besides, higher transport temperatures seem to correlate with a higher level of HSP70 during simulated transport. After 17 h transport, the HSP70 values gradually recovered, but did not return to the initial values, except at 15 °C. Cortisol showed a similar trend as HSP70, with significantly higher values at 27 °C than at other temperatures. At the end of transport, the cortisol concentration at 15, 18, and 21 °C recovered to the initial levels. HSP70 and cortisol increased with the transport temperature increase, which could illustrate that the tiger grouper had transport stress responses during simulated transport. A higher transport temperature could lead to unrecoverable stress response, resulting in death. Additionally, stress response was not significant, and fish could maintain the body balance through self-regulation [48].

### 3.3. Effect of Temperature on Antioxidant Enzyme of Tiger Grouper during Simulated Transport

Fish exposition to anoxia and hypoxia may result in oxidative changes, because oxygen consumption determines the levels of ROS generated, and also the antioxidant status [49]. Some clues could be given by an increase in activities of antioxidant enzymes under anoxic conditions [50]. Oxidative reactions are essential in normal metabolism of aerobic organisms, but ROS are produced during the oxidative metabolism, generating free radicals [51]. In a situation of oxidative stress, fish might show a typical reaction for ROS, involving lipoperoxidation (LPO), which can be quantified by an increase in TBARS levels. On the other hand, the deleterious effect of ROS can be balanced by the production of antioxidant defenses [52], such as CAT.

**Figure 2:**
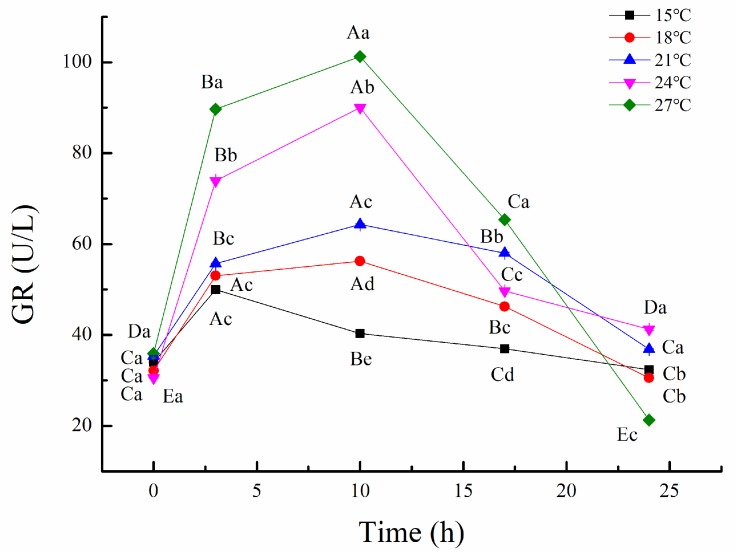
Effect of temperature on glutathione reductase (GR) of tiger grouper during simulated transport. Among different temperature transport groups, different small and capital letters indicate the results of Duncan’s test at different transport time. The same letters mean no significant difference (*p* > 0.05), while different letters mean significant difference (*p* < 0.05). Different letters without * = *p*-value < 0.05, different letters with ** = *p*-value < 0.01.

GR is an important indicator for evaluating the degree of oxidative stress [53]. Figure 2 showed that the GR levels increased first, and then decreased following transport time, reaching a peak level at 10 h, which was similar to cortisol and HSP70. It indicated that all tiger groupers were under different degrees of oxidative stress during early simulated transport. GR activity of tiger grouper transported at 27 °C was significantly higher than those at low temperatures, and the initial levels could not be recovered at the end of transport. In the 27 °C transport group, all of the tiger grouper suffered severe oxidation reaction, which caused irreversible damage and further affected the survival. However, GR activity recovered to the initial levels that were transported at 18 and 21 °C. Yan et al. [54] showed that fugu also had an oxidative stress reaction, due to the stressor of temperature. It led to HSP70 and GR activity increase. Therefore, a low temperature is suitable for tiger grouper transport, because of the decreased stress response and increased survival.

### 3.4. Effect of Temperature on Metabolic and Immune Enzyme Activity of Tiger Grouper during Simulated Transport

**Figure 3:**
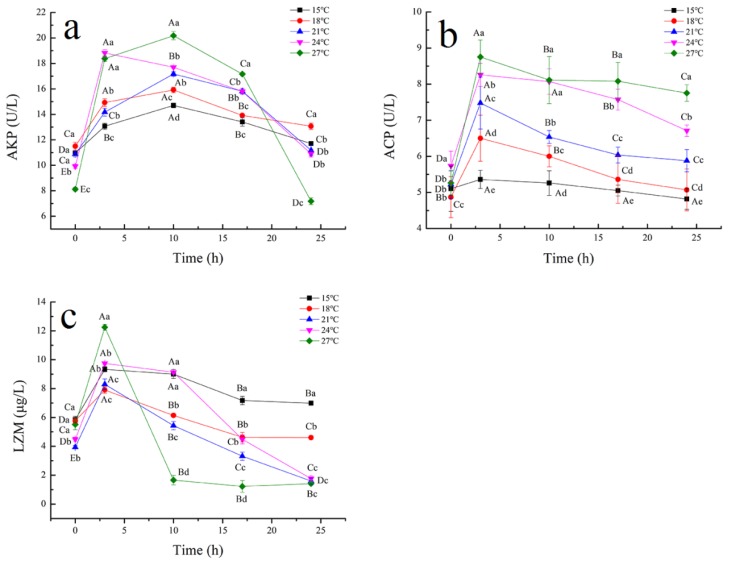
Effect of temperature on alkaline phosphatase (AKP) (**a**), acid phosphatase (ACP) (**b**), and lysozyme (LZM) (**c**) of tiger grouper during simulated transport. Among different temperature transport groups, different small and capital letters indicate the results of Duncan’s test at different transport time. The same letters mean no significant difference (*p* > 0.05), while different letters mean significant difference (*p* < 0.05). Different letters without * = *p*-value < 0.05, different letters with ** = *p*-value < 0.01.

Changes in the transport temperature not only induced a stress response in tiger grouper, but also lead to immune suppression. The main evaluation indexes of humoral immunity in fish include LZM [55,56]. AKP and ACP play important roles in phosphate hydrolysis in metabolic process and are key compounds in lysosomal digestion of invading organisms in the immune system [57]. AKP values significantly increased and reached a peak level at 10 h, and then decreased, as shown in Figure 3a. The final AKP values did not recover to the initial values at 24 °C, which meant that high temperature simulated transport could affect fish metabolism. However, the final AKP values could recover to the initial values at 15, 18, and 21 °C. ACP activities had similar trends to AKP; however, it reached a peak at 3 h (Figure 3b). The results suggested that the low temperature induced immune suppression response at the early stage of transport. As the first barrier of immunity, fish skin contains a large number of innate immune factors, as LZM in skin mucus, which mediates the protection against exogenous pathogen infection [58]. When the body is attacked by pathogens, LZM is secreted in the blood and mucus to eliminate these by activating blood cells and complement, and phagocytes in the liver and pancreas [59]. LZM values in the skin mucus of all tiger grouper firstly increased, and then decreased during simulated transport, as shown in Figure 3c. The highest value of LZM activity reached at 3 h, and then LZM activity plummeted to lower levels than the initial ones, indicating that the response of the innate immune system to temperature was at least partially suppressed. LZM activity of tiger grouper transported at 15 and 18 °C for 24 h could recover to the initial values, which indicate that fish damage can be decreased to the minimum at these temperatures.

### 3.5. Effect of Temperature on the Relative Expression of Immune Indexes of Tiger Grouper during Simulated Transport

**Figure 4:**
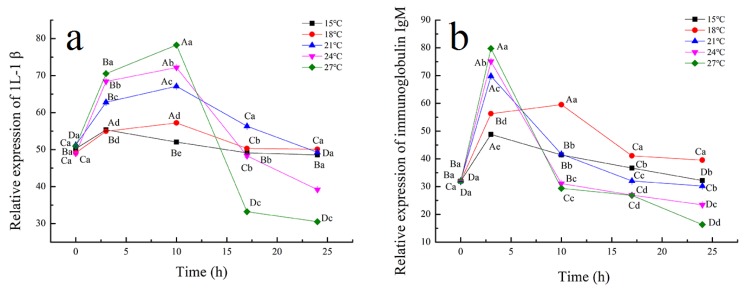
Effect of temperature on relative expression of 1L-1β (**a**) and IgM (**b**) of tiger grouper during simulated transport. Among different temperature transport groups, different small and capital letters indicate the results of Duncan’s test at different transport time. The same letters mean no significant difference (*p* > 0.05), while different letters mean significant difference (*p* < 0.05). Different letters without * = *p*-value < 0.05, different letters with ** = *p*-value < 0.01.

IgM and IL-1β are two important immune factors in fish immunity. IL-1β is one of the most important pro-inflammatory cytokines, which has a variety of immune response functions in viral infection, including the activation of innate immunity and regulation of adaptive immune response [60]. When affected by temperature stress, it can produce an acute-phase protein to activate innate immune regulation function [61]. IgM is one of the most important anti-pathogen antibodies, and the main immunoglobulin mediating humoral adaptive immunity of fish [62]. The expression levels of IgM and IL-1β significantly increased and came to a peak level at 3h and 10h, and then decreased, as shown in Figure 4. It was found that the expression of IgM in rainbow trout and Nile tilapia increased in a high-temperature environment, as compared to a low-temperature environment [63]. IgM expression in serum of tiger grouper transported at 27 °C was significantly higher than at other temperatures. The expression level at the end of transport was lower than the initial levels, which indicated that temperature stress made tiger grouper reach the threshold of innate immune ability, and unable to maintain the normal immune level through self-regulation. However, the tiger grouper transported at 15 °C could activate the innate immune system to maintain the immune balance and ensure the survival rate. This might be due to the dormancy induced in fish by this low-temperature.

### 3.6. Effect of Ascorbic Acid and β-1,3-Glucan Addition on Stress Responses of Tiger Grouper during Simulated Transport

**Figure 5:**
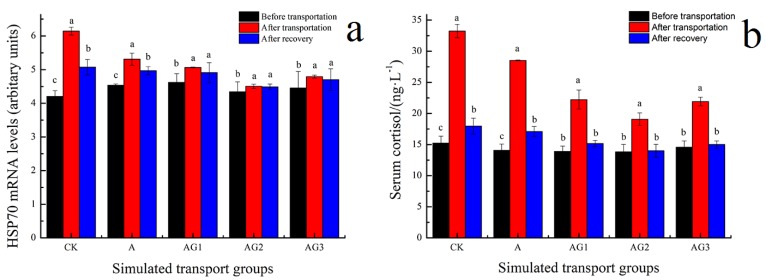
Effect of ascorbic acid and β-1,3-glucan addition on HSP70 (**a**) and serum cortisol (**b**) of tiger grouper during simulated transport. Among different treatments transport groups, different small letters indicate the results of Duncan’s test at different transport time. The same letters mean no significant difference (*p* > 0.05), while different letters mean significant difference (*p* < 0.05). Different letters without * = *p*-value < 0.05, different letters with ** = *p*-value < 0.01.

HSP70 and serum cortisol are indicators of transport stress response. The expression of HSP70 and the content of serum cortisol increased, and the treated samples were lower than that of CK, as recorded in Figure 5. The content of HSP70 and cortisol in A-G2 could recover to the initial level, which confirms the conclusion of Henrique et al. [64] that ascorbic acid addition can adjust the level of HSP70 and cortisol and, thus, regulate stress responses during fish transport. Therefore, ascorbic acid and β-1,3-glucan can be used to decrease transport stress responses for tiger grouper.

### 3.7. Effect of Ascorbic Acid and β-1,3-Glucan Addition on Relative Expression of Non-Specific Immune Indexes of Tiger Grouper during Simulated Transport

**Figure 6:**
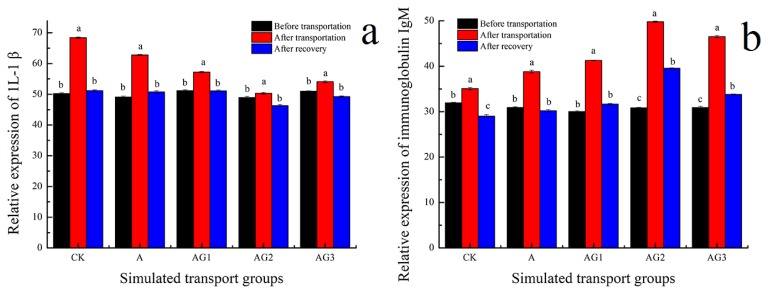
Effect of ascorbic acid and β-1,3-glucan addition on relative expression of 1L-1β (**a**) and IgM (**b**) of tiger grouper during simulated transport. Among different treatments transport groups, different small letters indicate the results of Duncan’s test at different transport time. The same letters mean no significant difference (*p* > 0.05), while different letters mean significant difference (*p* < 0.05). Different letters without * = *p*-value < 0.05, different letters with ** = *p*-value < 0.01.

Simulated transport usually induces stress responses that could lead to increase susceptibility to diseases. Immunostimulants can reduce the outbreak of diseases by facilitating the function of phagocytic cells, improving resistance to bacterial challenges [65]. The expression levels of IL-1β significantly increased, and then decreased, as shown in Figure 6a. IL-1β expression of CK was significantly higher than that of other simulated transport groups. From Figure 6b, the expression of IgM had a similar trend as that of IL-1β, and the expression of IgM in the A-G2 group was significantly higher than that of the other groups, which indicated that ascorbic acid and β-1,3-glucan addition could stimulate non-specific immune factors. Moreover, A-G2 had the highest relative expression of IgM, thus signifying that this concentration of β-1,3-glucan was most effective in this study, and it is considered suitable for transport.

### 3.8. Effect of Ascorbic Acid and β-1,3-Glucan Addition on Serum Biochemical Parameters of Tiger Grouper during Simulated Transport

Creatine kinase activity can act as an indicator of live fish metabolism. Increased creatine kinase suggests that muscle and kidney of fish have been damaged [66]. Creatine kinase activity of serum increased in all samples during simulated transport, and then returned to the initial level after recovery, as shown in Table 4. However, there was no significant difference in creatine kinase activity of A-G2 throughout the simulated transport, which indicated that ascorbic acid and β-1,3-glucan addition can effectively reduce the damage to kidneys.

Total protein (TP) and albumin (ALB) reflect the liver function. Albumin is synthesized by the liver, and plays a role as a carrier in the blood [67]. TP and ALB in the serum can accurately reflect the absorption and metabolism of the protein. The contents of TP and ALB in serum of CK were significantly higher than in other groups during simulated transport, as shown in Table 5. It suggests that the addition of ascorbic acid and β-1,3-glucan can improve the function of tiger grouper liver. The contents of TB and ALB in treated groups showed no significant difference throughout simulated transport. Moreover, TB and ALB of tiger grouper gradually returned to the initial level after transport and recovery.

Urea, creatinine, and uric acid (UA) reflect the renal function. Urea is the product of metabolism of nitrogen compounds, and also an important component in maintaining blood osmotic pressure [68]. At the end of transport, urea, creatinine and UA levels of treated samples decreased (Table 4), and these could not return to the initial level after recovery, which indicates damage that may have contributed to the fish majority during long-term transport.

### 3.9. Effect of Ascorbic Acid and β-1,3-Glucan Addition on Nutritional Indexes of Tiger Grouper during Simulated Transport

**Figure 7:**
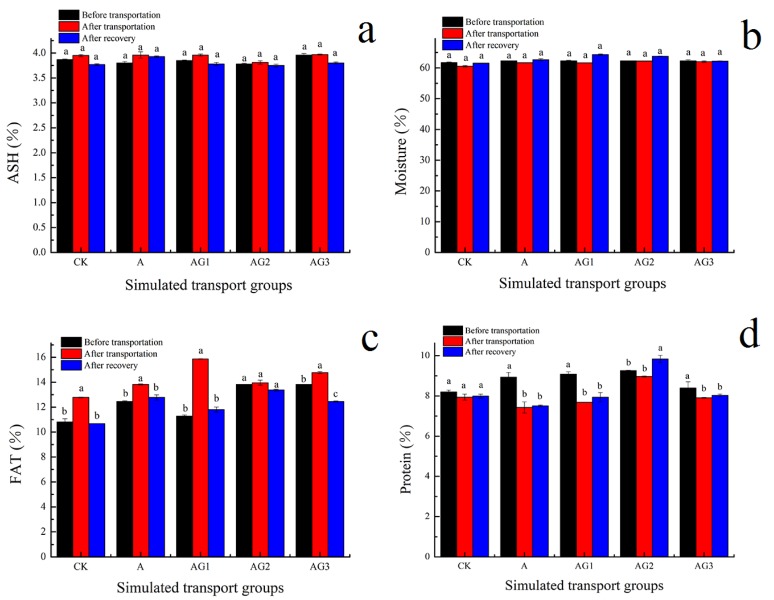
Effect of ascorbic acid and β-1,3-glucan addition on ash (**a**), moisture (**b**), fat (**c**) and protein (**d**) of muscle of tiger grouper during simulated transport. Among different treatments transport groups, different small letters indicate the results of Duncan’s test at different transport time. The same letters mean no significant difference (*p* > 0.05), while different letters mean significant difference (*p* < 0.05). Different letters without * = *p*-value < 0.05, different letters with ** = *p*-value < 0.01.

Figure 7 shows the changes in ash, moisture content, crude fat, and crude protein of tiger grouper during simulated transport. The contents of moisture and ash in all samples did not show obvious changes. Protein decreased, possibly because of stress related to transport and temperature change. Among all of the samples, the nutritional components of A-G2 showed no significant changes during simulated transport, which indicates that ascorbic acid and the G2 β-1,3-glucan concentration could effectively reduce the negative impact of transport and temperature changes on the nutritional indexes of tiger group.

### 3.10. Effect of Ascorbic Acid and β-1,3-Glucan Addition on Free Amino Acids of Tiger Grouper during Simulated Transport

**Figure 8:**
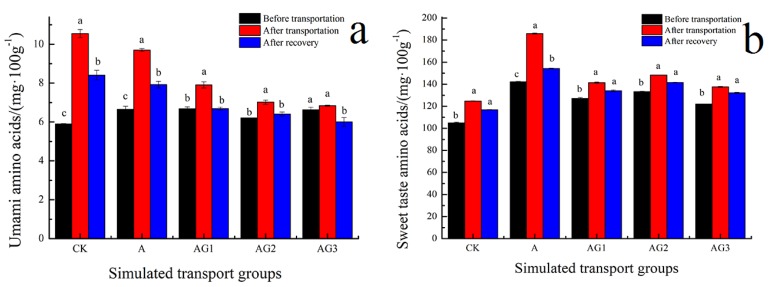
Effect of ascorbic acid and β-1,3-glucan addition on umami (**a**) and sweet (**b**) taste amino acids of tiger grouper during simulated transport. Among different treatments transport groups, different small letters indicate the results of Duncan’s test at different transport time. The same letters mean no significant difference (*p* > 0.05), while different letters mean significant difference (*p* < 0.05). Different letters without * = *p*-value < 0.05, different letters with ** = *p*-value < 0.01.

The total content of free amino acids of tiger grouper increased during simulated transport, and recovered to the initial level, as shown in Table 5. Transport stress could promote protein degradation, resulting in higher total free amino acids contents. It should be noted that there was no food for tiger grouper during simulated transport; therefore, transport stress could accelerate protein degradation and lead to nutrient content loss. However, the addition of ascorbic acid and β-1,3-glucan resulted in a reduction of free amino acid and thus probably slowed down the rate of protein degradation during simulated transport. This indicates moderation of the stress response. Free amino acids in the muscle tissue are usually related with different tastes, such as umami, sweetness, bitterness, and sourness. Umami amino acids include Asp and Glu, sweetness amino acids include Thr, Ser, Gly, Ala, and Pro [69]. Figure 8 shows the effect of ascorbic acid and β-1,3-glucan addition on umami and sweet taste amino acids of tiger grouper during simulated transport. In all samples, the amount of umami and sweet taste amino acids increased during simulated transport, due to transport stress response accelerated protein degradation. There was no significant difference in taste amino acids of A-G2 during simulated transport and recovery. The results indicate that ascorbic acid and the G2 β-1,3-glucan concentration can effectively reduce the changes of free amino acids during simulated transport. 

Nucleotides in aquatic products are of great significance for flavors [70]. IMP and AMP are the two main taste nucleotides in tiger grouper during simulated transport. IMP has a strong umami taste [71]. Table 6 shows the changes in IMP and AMP of tiger grouper during simulated transport. The results demonstrated that value of IMP was higher than 10. Therefore, IMP contributed most to the sweet and meaty flavor of tiger grouper. However, the content of AMP was obviously lower than IMP, and the TAV value of AMP was less than 1. IMP and AMP concentrations values were decreased during simulated transport and were able to recover to the initial levels. Different from the CK samples, the IMP and AMP concentrations in A-G2 samples were higher after recovery, and TAV value of IMP in A-G2 was 16.14, proving ascorbic acid and β-1,3-glucan addition could relieve flavor nucleotides degradation during simulated transport and recovery. 

## 4. Conclusions

The minimum tolerable temperature of tiger grouper transported by water is 15 °C, and could induce dormancy, thus resulting in reducing life activities. The activities of metabolic enzymes, cortisol, HSP70 transcription level, GR enzyme activity, IL-1β, and IgM transcription levels in tiger grouper serum at 15 °C were significantly lower than in groups transported at other temperatures. Anti-stress agents, including ascorbic acid and β-1,3-glucan was added in the transport water for tiger grouper, during simulated transport and recovery. Cortisol content, HSP70 transcription level, and immune index of tiger grouper serum in A-G2 were lower than in other groups. However, there was no significant difference in nutritional content, taste amino acids, and nucleotides of muscle tissue in A-G2 before and after transport. The addition of ascorbic acid and β-1,3-glucan could effectively reduce the stress response of tiger grouper and improve their immunity and survival. Besides, it did not lead to loss of nutritional valued and flavor.

## Figures and Tables

**Table 1:** Experimental design for exposure to anti-stress agents of live tiger grouper during simulated transport in water.

Samples	Anti-Stress Agent Addition
CK	Control
A	25 mg/L ascorbic acid
A-G1	25 mg/L ascorbic acid + 2.4 mg/L β-1,3-glucan
A-G2	25 mg/L ascorbic acid + 3.2 mg/L β-1,3-glucan
A-G3	25 mg/L ascorbic acid + 4.0 mg/L β-1,3-glucan

**Table 2:** Sequences of primers for Real-time PCR.

Target Gene	Primer Sequence (5′-3′)
HSP70	F: GACAAGAAGGTTGGGTCTGAAAGG
	R: GGTTGACCATGCGGTTGTCGAAATCT
IgM	F: GCCTCAGCGTCCTTCAGTTT
	R: TGGCGTCCCAGTCCTGTTTGC
IL-1β	F: AGGATGCCTGAGGGACTG
	R: GGTAATCGTCTCCAGATGTAA

**Table 3:** Survival rate of tiger grouper at different temperatures and survival time (%).

Temperature/°C	Keeping Alive Time/h						
	0	3	10	17	24	48	72
10	100	-	-	-	-	-	-
13	100	100	100	100	85	65	-
15	100	100	100	100	100	100	95
18	100	100	100	100	100	100	90
21	100	100	100	100	100	95	85
24	100	100	100	100	100	85	80
27	100	100	100	100	100	85	75
30	100	100	100	100	90	50	-

**Table 4:** Effect of ascorbic acid and β-1,3-glucan addition on serum biochemical and physiological of tiger grouper during simulated transport.

Transport	Samples	Creatine Kinase	Albumin	Total Protein	Uric Acid	Urea	Creatinine
Before transport	CK	848.50 ± 0.25a	11.00 ± 0.00a	41.50 ± 0.00a	13.00 ± 0.57b	2.50 ± 0.12a	17.00 ± 0.00a
	A	765.00 ± 0.13b	7.00 ± 0.23c	33.50 ± 0.71b	13.50 ± 0.23b	2.15 ± 0.16a	18.00 ± 0.31a
	A-G1	227.00 ± 0.66c	9.00 ± 0.16b	32.00 ± 0.36b	20.00 ± 0.06a	2.35 ± 0.11a	13.00 ± 0.06c
	A-G2	235.00 ± 0.57c	7.50 ± 0.03c	31.00 ± 0.00b	21.00 ± 0.71a	2.05 ± 0.08a	13.00 ± 0.06c
	A-G3	221.00 ± 0.08c	10.50 ± 0.00b	32.00 ± 0.08b	19.00 ± 0.35a	2.10 ± 0.06a	15.50 ± 0.24b
After transport	CK	1986.00 ± 0.58a	26.00 ± 0.21a	35.00 ± 0.32b	14.00 ± 0.03c	2.25 ± 0.00a	18.50 ± 0.17a
	A	1181.50 ± 0.97b	23.50 ± 0.00b	31.00 ± 0.06c	17.00 ± 0.21b	2.05 ± 0.28a	19.50 ± 0.00a
	A-G1	689.50 ± 0.69d	12.00 ± 0.36c	39.50 ± 0.00a	21.50 ± 0.19a	2.15 ± 0.14a	14.00 ± 0.00c
	A-G2	391.50 ± 0.73e	8.00 ± 0.42d	33.00 ± 0.57c	21.87 ± 0.09a	1.94 ± 0.03a	13.50 ± 0.25c
	A-G3	888.00 ± 0.29c	12.50 ± 0.00c	32.50 ± 0.69c	22.05 ± 0.15a	2.05 ± 0.00a	17.00 ± 0.14b
Recovery	CK	273.00 ± 0.93c	10.00 ± 0.32b	38.50 ± 0.53c	16.00 ± 0.32b	2.35 ± 0.00a	11.50 ± 0.27d
	A	769.00 ± 0.85a	6.50 ± 0.31c	46.00 ± 0.33a	16.50 ± 0.31b	2.40 ± 0.21a	20.00 ± 0.32a
	A-G1	267.50 ± 0.23c	13.00 ± 0.13a	43.50 ± 0.00b	17.00 ± 0.13b	2.30 ± 0.18a	18.00 ± 0.00b
	A-G2	225.00 ± 0.87d	6.50 ± 0.19c	32.50 ± 0.00d	16.50 ± 0.19b	2.30 ± 0.05a	14.00 ± 0.00c
	A-G3	390.50 ± 0.25b	11.00 ± 0.22b	34.50 ± 0.22d	22.00 ± 0.22a	2.25 ± 0.17a	18.50 ± 0.19b

Note: Among different treatments transport groups, different small letters indicate the results of Duncan’s test at different transport time. The same letters mean no significant difference (*p* > 0.05), while different letters mean significant difference (*p* < 0.05).

**Table 5:** Effect of ascorbic acid and β-1,3-glucan addition on the free amino acids of tiger grouper during simulated transport (mg/100 g).

Transport	Samples	Free Amino Acids								
		Asp *	Thr #	Ser #	Glu *	Gly #	Ala #	Val	Met	Ile
Before transport	CK	1.84 ± 0.25a	7.12 ± 0.25c	3.98 ± 0.17b	4.06 ± 0.22b	49.40 ± 0.49d	38.22 ± 0.71a	5.45 ± 0.31a	2.96 ± 0.26a	4.73 ± 0.34a
	A	1.76 ± 0.01a	11.44 ± 0.06b	4.87 ± 0.25a	4.89 ± 0.71a	86.09 ± 0.91a	34.33 ± 0.59b	4.30 ± 0.51b	2.07 ± 0.21a	3.01 ± 0.22b
	A-G1	1.65 ± 0.26a	10.93 ± 0.18b	3.77 ± 0.17b	5.03 ± 0.14a	75.24 ± 0.69b	31.29 ± 0.06c	5.98 ± 0.08a	2.56 ± 0.18a	3.97 ± 0.62a
	A-G2	1.79 ± 0.32a	12.14 ± 0.21a	4.05 ± 0.38b	4.42 ± 0.61b	71.25 ± 0.53b	39.78 ± 0.71a	5.09 ± 0.22a	2.13 ± 0.11a	4.17 ± 0.04a
	A-G3	1.84 ± 0.41a	11.03 ± 0.01b	4.97 ± 0.51a	4.79 ± 0.25a	66.42 ± 0.66c	33.86 ± 0.81b	4.97 ± 0.05a	2.44 ± 0.01a	3.57 ± 0.28b
After transport	CK	1.98 ± 0.03c	5.87 ± 0.45c	5.10 ± 0.05c	8.57 ± 0.07a	63.01 ± 0.21c	42.56 ± 0.66b	6.91 ± 0.06a	3.32 ± 0.28a	5.89 ± 0.41a
	A	3.37 ± 0.19a	9.85 ± 0.06b	7.62 ± 0.28a	6.33 ± 0.21b	124.35 ± 0.37a	38.03 ± 0.08d	5.13 ± 0.10b	2.62 ± 0.01b	2.50 ± 0.16c
	A-G1	2.38 ± 0.11b	10.01 ± 0.32b	7.99 ± 0.33a	5.53 ± 0.03c	75.44 ± 0.41b	40.24 ± 0.19c	6.02 ± 0.21a	2.78 ± 0.06b	3.11 ± 0.11b
	A-G2	1.93 ± 0.01c	10.93 ± 0.22a	6.46 ± 0.05b	5.09 ± 0.10c	78.03 ± 0.39b	46.51 ± 0.73a	5.67 ± 0.03b	2.61 ± 0.01b	3.55 ± 0.06b
	A-G3	1.70 ± 0.22c	10.90 ± 0.06a	5.48 ± 0.10c	5.14 ± 0.19c	74.29 ± 0.11b	41.22 ± 0.37c	5.13 ± 0.18b	2.48 ± 0.12b	3.71 ± 0.28b
Recovery	CK	1.82 ± 0.08a	7.89 ± 0.18c	4.77 ± 0.27a	6.59 ± 0.47a	58.98 ± 0.91c	40.03 ± 0.57b	6.84 ± 0.25a	2.38 ± 0.18a	4.84 ± 0.10a
	A	1.98 ± 0.10a	11.19 ± 0.27a	5.18 ± 0.27a	5.94 ± 0.31b	96.71 ± 0.43a	35.28 ± 0.09d	4.97 ± 0.33b	2.46 ± 0.02a	2.92 ± 0.16c
	A-G1	1.73 ± 0.02a	10.52 ± 0.04b	4.85 ± 0.03a	4.96 ± 0.80c	74.04 ± 0.09b	37.90 ± 0.18c	4.99 ± 0.09b	2.10 ± 0.11a	4.06 ± 0.71a
	A-G2	1.58 ± 0.16a	11.88 ± 0.11a	5.16 ± 0.18a	4.83 ± 0.33c	77.13 ± 0.36b	41.64 ± 0.78a	4.96 ± 0.18b	1.92 ± 0.02a	3.49 ± 0.39b
	A-G3	1.69 ± 0.22a	11.85 ± 0.65a	4.86 ± 0.20a	4.31 ± 0.57c	73.04 ± 0.74b	37.49 ± 0.07c	4.67 ± 0.21b	2.23 ± 0.18a	3.39 ± 0.27b
		**Leu**	**Tyr**	**Phe**	**Lys**	**His**	**Arg**	**Pro#**	**Total**	
Before transport	CK	7.52 ± 0.37a	3.22 ± 0.02a	2.88 ± 0.02a	28.54 ± 0.54a	3.86 ± 0.25a	7.86 ± 0.71a	6.27 ± 0.68a	177.91	
	A	7.73 ± 0.41a	1.45 ± 0.01c	1.57 ± 0.11c	21.89 ± 0.48c	3.55 ± 0.34b	5.38 ± 0.42c	5.49 ± 0.71b	199.82	
	A-G1	7.88 ± 0.68a	1.73 ± 0.22b	2.05 ± 0.18b	25.33 ± 0.71b	3.18 ± 0.33b	6.93 ± 0.74bc	5.93 ± 0.01a	193.45	
	A-G2	7.03 ± 0.31a	1.98 ± 0.17b	2.47 ± 0.17a	26.09 ± 0.31b	4.09 ± 0.06a	6.41 ± 0.11b	6.09 ± 0.39a	198.98	
	A-G3	7.97 ± 0.45a	2.06 ± 0.28b	1.99 ± 0.01c	23.45 ± 0.78c	3.74 ± 0.28a	7.07 ± 0.02b	5.77 ± 0.81ab	185.94	
Aftertransport	CK	7.83 ± 0.31a	3.76 ± 0.28a	3.46 ± 0.15a	31.35 ± 0.59a	4.30 ± 0.45a	10.74 ± 0.33a	8.18 ± 0.39a	212.83	
	A	7.76 ± 0.28a	1.34 ± 0.01c	2.28 ± 0.25b	24.39 ± 0.63c	3.63 ± 0.19b	4.99 ± 0.41d	6.07 ± 0.08c	250.26	
	A-G1	8.74 ± 0.41b	1.43 ± 0.12c	2.46 ± 0.06b	29.39 ± 0.71b	4.12 ± 0.06a	6.63 ± 0.71c	7.74 ± 0.73b	214.01	
	A-G2	7.01 ± 0.37a	1.55 ± 0.08c	2.49 ± 0.03b	27.83 ± 0.39b	4.23 ± 0.28a	6.69 ± 0.07c	6.41 ± 0.36c	216.99	
	A-G3	7.76 ± 0.63a	2.38 ± 0.19b	2.48 ± 0.11b	24.87 ± 0.23c	3.93 ± 0.09a	7.98 ± 0.20b	5.71 ± 0.51d	205.16	
Recovery	CK	6.82 ± 0.10b	2.95 ± 0.01a	1.79 ± 0.02b	22.19 ± 0.33c	3.60 ± 0.17a	6.59 ± 0.38a	5.19 ± 0.62c	183.27	
	A	6.98 ± 0.12b	1.52 ± 0.02b	1.98 ± 0.10ab	22.75 ± 0.62c	3.42 ± 0.31a	5.21 ± 0.67b	5.94 ± 0.17b	214.43	
	A-G1	7.65 ± 0.41a	1.67 ± 0.13b	2.30 ± 0.21a	26.91 ± 0.15a	2.75 ± 0.07b	6.87 ± 0.37a	6.79 ± 0.57a	200.09	
	A-G2	7.83 ± 0.57a	1.86 ± 0.03b	2.19 ± 0.09a	24.50 ± 0.31b	2.98 ± 0.35b	6.61 ± 0.65a	5.81 ± 0.08b	204.37	
	A-G3	7.23 ± 0.69a	1.44 ± 0.02b	2.26 ± 0.11a	19.59 ± 0.09d	3.64 ± 0.41a	6.55 ± 0.52a	4.94 ± 0.72c	189.18	

Note: Among different treatments transport groups, different small letters indicate the results of Duncan’s test at different transport time. The same letters mean no significant difference (*p* > 0.05), while different letters mean significant difference (*p* < 0.05). * represents umami amino acids; # represents sweet taste amino acids.

**Table 6:** Effect of ascorbic acid and β-1,3-glucan addition on nucleotides of tiger grouper during simulated transport in water.

Transport	Samples	IMP (mg/100 g)	TAV	AMP (mg/100 g)	TAV
Before transport	CK	269.18 ± 0.78d	10.77	13.24 ± 0.41ab	0.26
	A	273.40 ± 0.66c	10.94	14.69 ± 0.59a	0.29
	A-G1	271.94 ± 0.96cd	10.88	12.47 ± 0.65b	0.25
	A-G2	278.66 ± 0.55b	11.15	13.93 ± 0.32ab	0.28
	A-G3	284.39 ± 0.47a	11.38	13.54 ± 0.69ab	0.27
Aftertransport	CK	259.47 ± 0.84d	10.38	9.55 ± 0.57b	0.19
	A	268.06 ± 0.28c	10.72	11.23 ± 0.62ab	0.22
	A-G1	269.18 ± 0.71c	10.77	11.37 ± 0.71ab	0.23
	A-G2	275.33 ± 0.49b	11.01	12.99 ± 0.45a	0.26
	A-G3	280.91 ± 0.78a	11.24	10.81 ± 0.66b	0.22
Recovery	CK	273.57 ± 0.67e	10.94	14.43 ± 0.28a	0.29
	A	280.69 ± 0.71d	11.23	10.98 ± 0.33c	0.22
	A-G1	301.42 ± 0.54c	12.06	13.41 ± 0.19a	0.27
	A-G2	403.49 ± 0.66a	16.14	12.79 ± 0.25b	0.26
	A-G3	390.24 ± 0.91b	15.61	12.63 ± 0.36b	0.25

Note: Among different treatments transport groups, different small letters indicate the results of Duncan’s test at different transport time. The same letters mean no significant difference (*p* > 0.05), while different letters mean significant difference (*p* < 0.05).
